# Medical and Philosophical Intelligence

**Published:** 1818-12

**Authors:** 


### MEDICAL AND PHILOSOPHICAL INTELLIGENCE.

An eruptive varioloid disease, apparently similar to that which has appeared in the county of Derby, has lately been prevalent in Edinburgh and its neighbourhood, and has led to some interesting and important investigations, by Mr. Hennen and Dr Thomson, into the character small-pox has been frequently observed to assume since the use of vaccination, and particularly respecting the nature of the disease, apparently similar to variola, which affects persons who have had cow-pox. At present we can merely notice, that the opinions of the latter gentleman appear to coincide with those of Dr. Bent, related in the present Number of our Journal; but we shall consider this subject in the Historical Sketch of the Progress of Medicine during the last half-year, with which the ensuing Number of our Journal will commence.

---

The Edinburgh Medical and Surgical Journal contains a paper on the *use of Oil of Turpentine in Cases of Tænia*, by Mr. Hartle, of the General Hospital at Antigua. Medical men engaged in public practice are constantly endeavouring to call the attention of the profession to this useful and powerful remedy; but, unfortunately, the objections to it are such as to render the application of it in private practice very confined. Mr. Hartle administered two ounces of it, undiluted, as a dose, without finding it productive of any inconvenience, and with the most advantageous effects. The patients were private soldiers, who had been in the habit of drinking undiluted rum to a considerable extent. We rarely, in private practice, find patients who will bear more than two or three drachms as a dose, which, even when diffused with mucilage and copiously diluted, is often productive of distressing heat in the stomach, and a peculiar kind of intoxication. We must, however, remark, that, in the cases related by Dr. Fenwick (who first introduced the remedy to public notice), in the second volume of the Medico-Chirurgical Transactions, two or three ounces were ordinarily taken. Mr. Hartle correctly observes, that a large dose passes the bowels more quickly, and is less likely to produce inconvenience in the bladder or other distant parts, than a small one. We have observed that it acts most beneficially after the bowels have been well purged by an active cathartic. It is generally supposed that oil of turpentine is not effectual in the destruction of worms, unless it is taken undiluted: our experience does not lead us to concur with this opinion.

---

The same Journal contains a very elaborate Memoir, by Dr. Hamilton, written to prove that what is almost universally received as the true history of Lues (that is, the *lues venerea* of John Hunter), is not the true account; that Astruc does not trace the history of what we call Syphilis, but confounds it with another disease, viz.—Sibbens; and that sibbens, and not syphilis, was the disease which excited so much commotion throughout the medical world at the period to which we have alluded, and spread such devastation and dismay over the greater part of the habitable globe.

The manner in which that disease is stated to have been communicated, by all the authors who wrote on it at that period;—that is, by lying in the same bed, the use of the same towel, knife, glass, &c.—and the description of the symptoms of it which have been transmitted to us, are first adduced to support this opinion: in both which characters it resembles the accounts we have from the best authorities respecting sibbens. Sibbens appears to be now hardly known, except in the north of Scotland. *The inhabitants are unanimous in ascribing its introduction to the English soldiers who came among them under Oliver Cromwell.* This opinion Dr. Hamilton endeavours to support: Dr. Gideon Harvey, who wrote in 1666; Dr. John Wynell, in 1659; and Clowes, 1596; all concur in describing syphilis as being communicated by "kissing, shaking of hands, trying of gloves, &c.;" and state that, although it sometimes affected the genitals, yet it frequently appeared without having been preceded or accompanied with any disease of those parts; and that it generally spread through all the members of a family in which it made its appearance. Dr. H. remarks, that he wishes he could make it equally clear that, in all these cases, the sibben, the raspberry eruption, had been observed. But, he also observes, this is more difficult; for this particular eruption was called pustular by authors of a still earlier date. Sibbens has occasionally appeared in Europe at different periods in late times, and has generally spread with the rapidity, and committed the ravages, that the disease in question is described to have done by the older writers on it.

In the year 1800, a disease attracted attention in Dalmatia, affecting thousands with symptoms very like those of syphilis,—spreading by contact, without venereal congress. Dr. Cambieri considered it the same disease as the sibbens of Scotland; and Dr. Franck, of Vienna, sanctioned the opinion. The disease which in Norway is well known under the name of *radesyge*, and in Sweden under that of *saltfluss*, is by many regarded as the same with that which we are now considering. The continental authors of the later centuries give us numerous descriptions of it. Boerhaave, writing in 1727, is particularly strong: he makes unequivocal declarations, both in the preface to the Aphrodisiacus and in his Aphorisms. Nicolas de Blegny, practising at Paris in 1683, is, if possible, still more satisfactory. Dr. Hamilton then proceeds to produce particular inferences to prove that it was this disease which, at the siege of Naples in 1494, excited so much attention; and that it was this disease which was brought to Europe by Columbus,—which emanated from Hispaniola. He remarks, that it is, so far as he knows, universally believed that Astruc's History is the history of what we now call syphilis; and many of his observations undoubtedly have reference to this disease; and he believes that it was the venereal disease with which he was best acquainted: but it appears that he sometimes glides off, and describes the progress of sibbens.

Astruc gives the statements of historians, as well as of the medical men who observed the nature of the complaint in Hayti; who say, "It is in a manner hereditary to the natives; they not only contract it from venery, but it likewise breaks out upon them spontaneously."*—"All the natives are infected with this disease, than which there is none more contagious."†—"An inveterate itch was imported from the islands with the venereal disease."‡ The expressions of the physicians are not less strong.—"This disease is as familiar to them as the itch is to us, and is catched by infection in the same manner."§—"The disease, being infectious, soon spread to the soldiers who kept company with the natives: it was by them (the soldiers) ascribed to their hardships."|| Dr. Hamilton then follows the disease into Europe. When the followers of Columbus returned with him from the West-Indies, they went to Barcelona: "immediately the whole city was seized with the disease; it spread all over it. They imagined it owing to some peculiar state of the atmosphere." From Spain it spread throughout Europe; and the manner of its propagation is so notorious, (continues Dr. H.) that he feels himself called upon particularly to describe it. This he does from the Aphrodisiacus of Luisinus,—a compilation in which all the works of any value on the subject, up to the year 1566, is contained: but our limits will not permit us to follow Dr. Hamilton in his researches in this respect,—we must conclude with his general observations on them.

"Many of the authors I have already quoted give, for example, such relations as this:—They were called to a family, into which the disease had been introduced by a stranger,—say a child. This child communicated it to the mother, who was perhaps suckling it. The mother transmitted it to her husband,—he to his son, a boy of six years of age,—he again to his brother, of ten,—they together to their sister, a girl of 12 or 14, and so on; and these are affected without the genitals participating. Many instances, I say, of this sort are given, and I am prevented from particularly detailing them, merely from a fear of too far exceeding the limits of my paper. These minute details, while they show the extent and mode of communication, also point out the nature and symptoms of the affection.

"I am willing to allow that true syphilis may sometimes propagate itself without venereal intercourse. But this is a rare occurrence, and happens so seldom, that, if the statements I have quoted and referred to are entitled to any kind of credit, they altogether preclude the possibility of the disease under consideration being syphilis.

"But again, it may be said, granting that it is not syphilis, how is it satisfactorily proved that it was not leprosy or itch?—that there was a cachectic state of the constitution at all resembling that produced by syphilis?—in short, that it was sibbens?

"With regard to leprosy, I would remark, that it is now agreed, among the respectable authors of the present day, that this disease was at no time mistaken for the venereal.

---

* J. Baptist du Tertre, Astruc, p. 79.

† F. Guicciardini, u. s. ‡ Diaz. p. 7, u. s.

§ F. Lapez, in p. 79. || J. Montanus, in p. 79.

"Moreover, the very minute descriptions that are given by many of the authors altogether preclude the supposition of its being mistaken for leprosy or itch, or any disease merely cutaneous. Many of the descriptions would do honour to the accuracy of our own time. They describe it as a disease, not merely affecting the skin, but the throat also,—the bones—the hair—the whole constitution. They clearly describe a disease, in which much of that derangement produced by the venereal poison exhibits itself."

---

A very interesting subject, as a *Prize Question*, has been proposed by the Medical Circle (late the Academy of Medicine) of Paris, to "determine the influence of pathological anatomy (morbid anatomy) on the progress of medicine in general, and particularly on the diagnosis and treatment of internal diseases?" The premium will be a gold medal of the value of 300 francs; and will be decreed in an extraordinary public sitting, which will take place in October 1819. The Memoirs are to be written either in French or Latin, and bear, in the usual manner, a motto similar to one in a sealed packet, containing the name of the author. They are to be sent, free of expense, before the end of July 1819, to M. le Dr. Chardel, secrétaire général du Cercle Medical, rue Cassette, No 23, Paris.

The ordinary members of the Society are alone exempt from the privilege of contesting it.

The Society of Medecine of Marseilles have proposed, as the subject for dissertation for the following year, the following questions:—

"1st.—What are the diseases of the uterus which are liable to be confounded with cancer and ulceration of that organ?

"2d.—What are the characteristics by which they may be decidedly distinguished?

"3d. What are the curative or palliative measures that experience has proved to be most efficacious?"

The Society desires that the candidates will make clinical observations, and *post mortem* examinations, the bases of their observations. The Memoirs are to be written legibly in French or Latin, and sent, with the usual forms, to M. Trucy, docteur en medécin, secrétaire général de la Société de Medécine de Marseille, before the 1st of July, 1819. The premium is a gold medal, of the value of 300 francs.

The Royal Medical Society of Bourdeaux has proposed the following:—"What are the results of too rapid growth *(d'un accroissement trop rapide)*? What are the means adapted to moderate its progress, if they become injurious, and to remedy the accidents which ensue from it?"

The Society expects that candidates will not give way to reflections drawn from subtle hypotheses; it desires a Memoir containing positive facts, which may be incontestibly acknowledged by practical medicine. The Memoirs are to be sent in the form and manner mentioned respecting the preceding ones, to the secretary of the Society, before July 1, 1819. The premium is 300 francs.

### REPORT OF DISEASES.

THE advice of HIPPOCRATES—Ωστε ἐς πόλιν ἐπειδὰν ἀφίκηταί τις, ἧς ἄπειρός ἐστι, διαφροντίσαι χρὴ τὴν θεσιν αὐτέης, ὅκως κέεται καὶ πρὸς τὰ πνεύματα, καὶ πρὸς τὰς ανατολας· τοῦ ἡλίου· οὐ γὰρ τωὐτὸ δυναται, ἥτις πρὸς βορέην κέεται, καὶ ἥτις πρὸς νότον· οὐδ᾽ ἥτις πρὸς ἥλιον ἀνίσχοντα, οὐδ᾽ ἥτις πρὸς δύνοντα,—is particularly applicable to this extensive metropolis at the present season of the year.

A marked distinctness of character may be observed in the febrile diseases prevalent in the different districts: in the northern and eastern, they are, for the most part, characterized by evident and active local inflammation; in the western and interior parts of the city, general febrile commotion, debility, great derangement of the nervous system,—in a word, the combination of symptoms constituting what is usually termed typhous fever,—is generally observed: a distinctness of character, doubtless, arising from the different state of the air, the influence of winds, and frequency of changes of the temperature of the atmosphere, which may be observed between them. In our last Report we spoke of puerperal fever having been witnessed in London more frequently than usual: this continues to exist, but is less prevalent than at the period to which we have alluded; and it has been almost universally confined to the north-eastern suburbs. It has been almost exclusively treated with copious abstraction of blood, active purgatives (particularly of large doses of calomel), and has terminated fatally in but a very few instances. Many intelligent and experienced practitioners are still inclined to believe the disease to be contagious; but we have never been able to witness a decisive instance in which it has appeared to have been communicated in this manner. The less important febrile disorders have been, as usual at this period, catarrh, asthma, peritoneal inflammation, and acute rheumatism; the latter has been more than ordinarily prevalent. The effects resulting from the mode of practice in this disease to which we alluded in our last Report, which have occurred to our knowledge, make us regret exceedingly that it is so seldom employed by general practitioners. The little benefit that has accompanied the use of it, is frequently mentioned as the cause for this neglect; but we are disposed to attribute this to a want of that adroitness and attention which are necessary in order to obtain advantageous results from its application.

Chronic inflammation of the mucous membrane of the bronchiæ is rather frequent. In several cases of this kind, which have been of long continuance, where depletion has been freely employed, and the patients have fallen into a state of great debility, accompanied with peculiar depression of spirits, fever simulating hectic, and copious morning sweats; and in which a large quantity of adhesive phlegm, without admixture of pus, has been expectorated; we have seen very advantageous effects result from the use of muriatic acid:—the sallow bluish tinge of the skin, and dilated state of the veins, have quickly disappeared; the appetite returned, and the local disease been considerably, and in many cases entirely, relieved by it. To produce these effects, the muriatic acid must be administered to the extent of one drachm in the twenty-four hours, diluted in a quart of water, with an addition of sugar and a small proportion of brandy.

The cases of chronic inflammation of the heart, to which we alluded in our last Report, have done so well under the old plan of treatment, that we have not yet been disposed to employ the laurel-water. Considerable benefit has evidently resulted from the production of a rash over the whole of the left side of the thorax, by means of the tartar-emetic ointment, in these cases. On ceasing to induce it, from disinclination of the patients to undergo this (rather distressing) part of the curative treatment, the symptoms of the disease have become more severe; and a recurrence of palpitation, anxiety, &c. has appeared during the suspension of its use, in instances where those alarming symptoms had been long absent.

### METEOROLOGICAL JOURNAL,

### By Messrs. WILLIAM HARRIS and Co. 50, Holborn, London.

*From the 20th October to the 19th December, inclusive.*

| Day of Month. | Moon. | Rain gauge | Therm. | | | Barom. | | De Luc's Hygrom. Dry | Damp | | Wind. | | Atmospheric Variation. | | |
|---|---|---|---|---|---|---|---|---|---|---|---|---|---|---|---|
| Oct. | | | | | | | | | | | | | | | |
| 20 | | | 58 | 65 | 53 | 30·05 | 30·17 | | 16 | 17 | SE | SSE | Fog | Fine | |
| 21 | | ,05 | 54 | 60 | 53 | 30·09 | 30·05 | | 20 | 19 | E | ENE | Fog | Fine | |
| 22 | | | 54 | 59 | 51 | 29·97 | 29·95 | | 17 | 17 | E | E | Fine | | |
| 23 | | | 53 | 60 | 49 | 29·00 | 29·97 | | 17 | 19 | E | E | Clo. | Fine | |
| 24 | | | 61 | 60 | 47 | 30·99 | 30·00 | | 20 | 13 | NE | NNE | Fine | Clo. | Rain |
| 25 | | ,15 | 55 | 63 | 43 | 29·00 | 29·97 | | 21 | 17 | E | E | Fine | | |
| 26 | | | 47 | 61 | 43 | 30·00 | 30·01 | | 19 | 21 | NE | NE | Fine | | |
| 27 | | | 47 | 59 | 49 | 30·10 | 30·05 | | 20 | 20 | E | E | Fine | | |
| 28 | | | 54 | 63 | 50 | 30·25 | 30·17 | | 16 | 18 | SW | SW | Fine | | |
| 29 | | | 53 | 64 | 50 | 30·25 | 30·31 | | 18 | 17 | SW | SW | Fog | Fine | |
| 30 | | ,02 | 52 | 61 | 48 | 30·00 | 30·17 | | 15 | 14 | SW | SSW | Fog | Fine | |
| 31 | | ,03 | 50 | 60 | 50 | 30·95 | 30·00 | | 12 | 14 | SW | WSW | Fog | Fine | |
| Nov. | | | | | | | | | | | | | | | |
| 1 | | | 53 | 61 | 48 | 29·85 | 29·92 | | 16 | 19 | W | W | Fog | Fine | |
| 2 | | | 53 | 59 | 50 | 29·79 | 29·87 | | 15 | 10 | W | W | Clo. | Fine | |
| 3 | | | 54 | 60 | 47 | 29.71 | 29·75 | | 9 | 10 | SW | SW | Fog | Fine | |
| 4 | | | 49 | 61 | 48 | 29·77 | 29·69 | | 7 | 5 | WNW | NW | Fine | | |
| 5 | | | 50 | 59 | 44 | 29·77 | 29·81 | | 5 | 9 | WNW | W | Fine | | |
| 6 | | | 48 | 55 | 42 | 29·80 | 29·85 | | 11 | 10 | SW | SW | Fine | | |
| 7 | | | 46 | 56 | 40 | 29·88 | 29·94 | | 10 | 10 | SSW | SW | Fine | | |
| 8 | | | 45 | 56 | 39 | 29·90 | 29·94 | | 12 | 14 | E | E | Fine | Clo. | |
| 9 | | | 43 | 55 | 40 | 29·94 | 30·01 | | 13 | 12 | SE | E | Rain | Fine | Clo. |
| 10 | | ,09 | 14 | 53 | 41 | 30·00 | 29·95 | | 17 | 14 | S | SW | Fog | Rain | Clo. |
| 11 | | ,07 | 45 | 55 | 40 | 29·87 | 29·89 | | 14 | 13 | SW | W | Fog | Clo. | Rain |
| 12 | | ,10 | 46 | 59 | 43 | 29·86 | 29·79 | | 15 | 17 | W | SW | Rain | Clo. | |
| 13 | | ,08 | 46 | 57 | 45 | 29·70 | 29·63 | | 16 | 18 | SSE | SE | Fog | Rain | Clo. |
| 14 | | ,03 | 49 | 58 | 47 | 29·57 | 29·65 | | 21 | 20 | SE | E | Rain | | |
| 15 | | ,17 | 49 | 55 | 45 | 29·64 | 29·60 | | 11 | 16 | SSE | SE | Fine | Rain | |
| 16 | | ,05 | 50 | 54 | 47 | 29·63 | 29·70 | | 15 | 12 | SE | SE | Rain | | Clo. |
| 17 | | ,05 | 50 | 52 | 47 | 29·76 | 29·98 | | 17 | 16 | SE | SE | Clo. | Fine | Clo. |
| 18 | | | 48 | 48 | 45 | 30·04 | 30·07 | | 16 | 16 | SE | SE | Fog | Clo. | Fine |
| 19 | | | 49 | 52 | 40 | 30·07 | 30·01 | | 15 | 16 | SE | SE | Fog | Clo. | Fine |

The quantity of rain fallen in the month of October is 1 inch and 43-100ths.

